# Editorial: In Search of Mechanisms: Genes, Brains, and Environment in Aggressive Behavior

**DOI:** 10.3389/fpsyt.2021.643747

**Published:** 2021-03-08

**Authors:** Marieke Klein, Noèlia Fernàndez-Castillo

**Affiliations:** ^1^Department of Human Genetics, Donders Institute for Brain, Cognition and Behaviour, Radboud University Medical Center, Nijmegen, Netherlands; ^2^Department of Psychiatry, University of California, San Diego, La Jolla, CA, United States; ^3^Departament de Genètica, Microbiologia i Estadística, Facultat de Biologia, Universitat de Barcelona, Barcelona, Spain; ^4^Centro de Investigación Biomédica en Red de Enfermedades Raras (CIBERER), Madrid, Spain; ^5^Institut de Biomedicina de la Universitat de Barcelona (IBUB), Barcelona, Spain; ^6^Institut de Recerca Sant Joan de Déu (IR-SJD), Esplugues de Llobregat, Barcelona, Spain

**Keywords:** aggression, genetics, epigenetics, animal models, biomarkers, hormones, GWAS, EWAS

Aggression is a behavior with evolutionary origins as a heritable trait essential for survival and fitness. However, in today's society it is often both destructive and maladaptive, as context-inappropriate aggression can cause harm to society, families, and individuals. Aggression may be defined as hostile behavior with the intention of inflicting damage or harm, but it is a behaviorally and etiologically complex phenomenon, including traits such as rule breaking, violence, delinquency, and criminality. The heritability of human aggression has been estimated at around 50% and its complex genetic architecture interacts with environmental factors. Such a multifactorial phenotype calls for novel and innovative methodological approaches but also for new hypotheses about underlying biological mechanisms. The diverse contributions to this Research Topic reflect various approaches and opinions in the field of aggression (epi)genetics and could be understood as innovative responses to the challenges in the research on multifactorial phenotypes. This Research Topic focuses on the identification of the underlying mechanisms of aggressive behavior at multiple levels of (epi)genetic, neurobiological and cognitive complexity. By integrating different aspects of epidemiological research, molecular genetics, functional animal models, neurobiological and psychological aspects this Research Topic aims to shed some light on the differential etiological pathways of aggression.

Unraveling the genetic basis of aggressive behavior, so far unclear, would help to understand this complex behavior and its co-occurrence with other disorders or traits. Tielbeek and Boutwell explored the genetic correlations of aggression with health and physiological traits, obtaining no significant findings, which may suggest that these phenotypic associations do not have a genetic cause. In this perspective the authors emphasize the need for larger samples, dissect homogeneous subtypes, and explore causality (Tielbeek and Boutwell). Vaht et al. inspected the association of several variants in the gene *RBFOX1*, which were previously associated with aggression. Although no significant findings were observed for aggressiveness, some variants were associated with neuroticism, extraversion as well as alcohol use disorder, and propose that future studies should consider personality traits or substance use in aggression (Vaht et al.).

Contextual personality influences aggressive behavior and it is important to elucidate which factors trigger an aggressive response. Weidler et al. used the Taylor Aggressiveness Paradigm (TAP) to clarify the role of physical and non-physical provocation on reactive aggression. They found that provocation is a strong predictor for aggression and that loss and frustration in competition triggers aggression. They also observed that women react less aggressively than males under low provocation and hesitant to punishment, but similar when provocation was prolonged or high (Weidler et al.).

Increased aggressive behavior has been associated with neuropsychiatric disorders, such as attention-deficit/hyperactivity disorder (ADHD). Meijer et al. studied how the interplay between genetic and environmental factors may affect ADHD and related traits, such as impulsivity and callousness, via DNA methylation. They performed epigenome-wide association studies (EWAS) but did not identify any significant epigenome-wide single CpG sites or regions associated with impulsive and callous traits. However, hypermethylation of *APOB* and *LPAR5* might be associated with persistent ADHD, suggesting an involvement of fatty acid metabolism. This may provide new hypotheses for research into the molecular mechanism of ADHD and related traits (Meijer et al.).

Animal models are commonly used to study neurobiological mechanisms underlying aggressive traits. van Heukelum et al. investigated how the distribution of parvalbumin (PV) and somatostatin (SOM) interneurons across the anterior cingulate cortex (ACC) and midcingulate cortex (MCC) differentially predicts aggression and social withdrawal in BALB/cJ mice. Their results suggest that a specific balance of inhibitory control across the ACC may be required to successfully regulate complex behaviors such as aggression and social contact (van Heukelum et al.). Using the same mouse strain, Jager et al. investigated dose-dependent effects of methylphenidate (MPH). This psychostimulant increases monoamine concentrations and can be an effective treatment in reducing aggression in patients with conduct disorder (CD) and ADHD. Their results support a role for MPH in the regulation of anxiety, fear processing, and aggression in BALB/cJ mice, with the latter effect in a dose-dependent manner (Jager et al.). More detailed knowledge of the efficacy of MPH administration can help in understanding its potential impact in the clinical management of conduct problems, aggressive behavior and other ADHD-related phenotypes.

The identification of biomarkers is of great interest, as these may serve as potential diagnostic and predictive instruments in personalized medicine. Hagenbeek et al. present the first metabolomics study by investigating the association between urinary metabolites and neurotransmitter ratios and aggressive behavior in children. In the discovery phase, six biomarkers were significantly associated with childhood aggression, converging in suggestive evidence for associations of childhood aggression with metabolic dysregulation of neurotransmission, oxidative stress, and energy metabolism (Hagenbeek et al.). Finally, Vaeroy et al. reviewed the literature to discuss the role of autoantibodies reactive with stress-related hormones, such as adrenocorticotropic hormone (ACTH), oxytocin, or arginine vasopressin. Altered levels in plasma of autoantibodies for these hormones have been found in individuals with antisocial behavior, prisoners, or with increased aggression, especially ACTH. These hormones modulate the HPA axis, which is hypothesized to mediate aggression in response to stress, although further studies should clarify the origin and effects of these autoantibodies (Vaeroy et al.).

This Research Topic highlights the importance of the need for further studies with increased sample sizes to advance the field of genetics and epigenetics of aggression, and the inclusion of other related traits or behaviors relevant for aggression (personality traits, externalizing behaviors, ADHD, etc.). On the other hand, it has helped to advance our understanding of the biological mechanisms underlying aggressive behavior, highlighting relevant neuronal circuits (in mice), biomarkers, and possibly effective treatments, which may be of help from a clinical perspective.

## Author Contributions

All authors listed have made a substantial, direct and intellectual contribution to the work, and approved it for publication.

## Conflict of Interest

The authors declare that the research was conducted in the absence of any commercial or financial relationships that could be construed as a potential conflict of interest.

